# An adjuvant free mouse model of oral allergenic sensitization to rice seeds protein

**DOI:** 10.1186/1471-230X-11-62

**Published:** 2011-05-23

**Authors:** Xiao-Wei Chen, Ken Wan-Keung Lau, Fan Yang, Samuel Sai-Ming Sun, Ming-Chiu Fung

**Affiliations:** 1 Biology Program, School of Science Life Sciences, The Chinese University of Hong Kong, Shatin, Hong Kong SAR, China

## Abstract

**Background:**

Rice is commonly known as a staple crop consumed worldwide, though with several rice proteins being reported for allergic properties in clinical studies. Thus, there is a growing need for the development of an animal model to better understand the allergenicity of rice proteins and the immunological and pathophysiological mechanisms underlying the development of food allergy.

**Methods:**

Groups of BALB/c mice were sensitized daily with freshly homogenized rice flour (30 mg or 80 mg) without adjuvant by intragastric gavage. In addition, the mice were challenged with extracted rice flour proteins at several time points intragastrically. Hypersensitivity symptoms in mice were evaluated according to a scoring system. Vascular leakage, ELISA of rice protein-specific IgE, histopathology of small intestine, and passive cutaneous anaphylaxis were conducted on challenged mice.

**Results:**

An adjuvant free mouse model of rice allergy was established with sensitized mice showing increased scratching behaviors and increased vascular permeability. Rice protein-specific IgE was detected after eighteen days of sensitization and from the fifth challenge onwards. Inflammatory damage to the epithelium in the small intestine of mice was observed beyond one month of sensitization. Passive cutaneous anaphylaxis results confirmed the positive rice allergy in the mouse model.

**Conclusions:**

We introduced a BALB/c mouse model of rice allergy with simple oral sensitization without the use of adjuvant. This model would serve as a useful tool for further analysis on the immunopathogenic mechanisms of the various rice allergens, for the evaluation of the hypersensitivity of rice or other cereal grains, and to serve as a platform for the development of immunotherapies against rice allergens.

## Background

Rice, a major staple crop, is consumed by more than half of the world's population, especially in East Asia [1]. Multiple investigations show that rice may lead to a certain degree of hypersensitivity. Direct contact with raw rice, inhalation of rice pollen or vapor from boiling rice, and oral ingestion of cooked rice are a few defined routes that may trigger rice allergy [2-4]. Since the first identification of allergenicity from the rice protein fractions containing albumin, globulin and glutelin [5], rice allergy has been reported in many countries around the world, such as Japan, Malaysia, Thailand, Indonesia, and some European countries like Finland, France, Sweden, Denmark, Estonia, Lithuania, and Russia [2].

Rice hypersensitivity has been mainly defined as IgE-mediated reactions which ranged from mild urticaria to anaphylactic reactions [6-8]. There are also some evidences suggesting that rice is the most common solid food causing food protein-induced enterocolitis syndrome (FPIES), which is defined as a non-IgE-mediated gastrointestinal food hypersensitivity disorder [9,10]. Although several rice allergens have been identified, the immunopathogenic mechanisms responsible for IgE- and cell-mediated systemic reactions are not well characterized. Murine model has been considered a good experimental system for studying the pathological mechanisms of food allergy [11,12]. However, such model of rice allergy has yet to be established and the mechanisms for hypersensitivity caused by rice proteins await detailed elucidation.

The purpose of this study is to establish a rice allergy mouse model using a simple protocol: daily sensitization with rice flour through the oral route without adjuvant to mimic the natural human exposure to ingested rice, as well as the experience of multiple intragastric challenges caused by rice total protein hypersensitivity.

## Methods

### Rice protein (RP) extraction and electrophoresis

Rice (*Oryza sativa *L. subsp. *japonica*) proteins extraction was conducted as described by Ju and Hettiarachchy [13], with little modifications. Briefly, after defatted with n-hexane (Fairfield, OH, USA), dried rice flour was processed by four volumes of distilled water, 5% NaCl (Sigma-Aldrich, St Louis, MO, USA), 0.02 M NaOH (Sigma-Aldrich), and 70% ethanol (Scharlau Chemie SA, Barcelona, Spain) for the extraction of albumin, globulin, glutelin and prolamin, respectively. All extractions were performed by shaking at 20°C for 4 h and centrifuged at 17,700 g with the exception that glutelin was extracted by shaking at 20°C for 30 min. Each extraction was repeated twice. The supernatants were collected and filtered. Albumin, globulin, and glutelin were precipitated by adjusting the pH to match their corresponding isoelectric points. Prolamin was precipitated by adding double volume of acetone (VWR Scientific, Suwanee, GA, USA) to the supernatant. The precipitated RPs (albumin, globulin, glutelin, and prolamin) were washed twice with distilled water, adjusted to pH 7.0, freeze-dried and stored at 4°C. Protein concentrations were determined by following the Kjeldahl's method (AAAC, 1983). Sodium dodecyl sulfate - polyacrylamide gel electrophoresis (SDS-PAGE) was carried out under reducing conditions on 12.5% polyacrylamide gel. Proteins were visualized with Coomassie Blue staining (Bio-Rad Laboratories, Hercules, CA, USA).

### Rice sensitization and challenge protocol

Female BALB/c mice (6 - 8 weeks old) were purchased from the Laboratory Animal Services Centre of the Chinese University of Hong Kong (Hong Kong SAR, China). The animals were maintained on rice-free diet and kept in a temperature controlled room (23 ± 2°C) with a 12 h light: 12 h dark cycle. All animal procedures were approved by the Animal Experimentation Ethics Committee of the Chinese University of Hong Kong, in accordance with the Department of Health (Hong Kong) guidelines in Care and Use of Animals. All experiments were performed under licenses granted from the Hong Kong Special Administrative Region Government.

**Figure 1:**
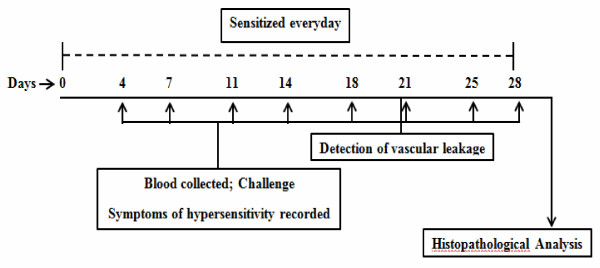
**Schematic representation of the sensitization and challenge in this study**. Groups of mice (n = 6) were daily intragastically (i.g.) sensitized with 30 mg or 80 mg freshly homogenized rice flour. Challenges were performed at day 4, 7, 11, 14, 18, 21, 25, and 28 after sensitization in each experimental group. Before each challenge, mice were bled from the submandibular veins. The symptoms of hypersensitivity were recorded 1-1.5 h after challenge. Vascular leakages were detected before the 6th challenge. The mice were sacrificed 4 days after the last challenge (day 28), and histopathological analysis was performed. The control group mice were orally sensitized and challenged with PBS.

Three groups each with 6 BALB/c mice were included in this study. In two experimental groups, mice were daily sensitized by intragastic gavage (i.g.) of 30 mg or 80 mg freshly homogenized rice flour. Upon each specified time point (day 4, 7, 11, 14, 18, 21, 25, and 28 after the course of sensitization), mice were orally challenged with 30 mg of the extracted rice flour proteins. Control mice received an equal volume of phosphate buffered saline (PBS) for daily sham sensitization and challenge (Figure 1). All materials used to feed the mice were homogenized or dissolved in 1 ml PBS solution. Before each challenge, mice were bled from the submandibular veins [14]. Sera were collected and subsequently stored at -80°C until use.

### Assessment of hypersensitivity responses

Symptoms of hypersensitivity were evaluated within 1-1.5 h after each challenge using a scoring system previously reported and scored as followed: 0 = no symptoms; 1 = scratching and rubbing around the nose and head; 2 = puffiness around the eyes and mouth, pilar erecti, reduced activity, and/or decreased activity with increased respiratory rate; 3 = wheezing, labored respiration, and cyanosis around the mouth and the tail; 4 = no activity after prodding or tremor and convulsion; and 5 = death [15].

### Detection of vascular leakage

To assess the vascular permeability, 3 to 5 mice from each group received 100 μl of 0.5% Evan's blue Dye (Sigma) through tail vein injection immediately before the sixth intragastric RP challenge. Footpads of mice were recorded for signs of vascular leakage (visible bluing) within 1-1.5 h after dye/antigen administration.

### Passive cutaneous anaphylaxis test

Pooled sera were obtained from each group of mice experienced different sensitization. Passive cutaneous anaphylaxis (PCA) test were performed as what has been previously described with slight modifications [15,16]. The abdomens of four naïve mice were shaved and four injection sites on each mouse were marked (three repeated experimental sites and one negative control site). On the first day, mice were injected with 50 μl heated sera (56°C for 3 hours) from the 80 mg rice flour proteins sensitized mice group or unheated sera (undiluted) from each group respectively. Control mice received an equal amount of naïve serum. After twenty-four hours, mice were re-injected at the same sites again with 50 μl pooled same sera as the first injection. After an additional 3 h, mice were injected intravenously with 100 μl of 0.5% Evan's blue dye followed by intradermal injection of 50 μl rice flour protein (2 mg/ml). PBS injection was applied to the control site of each mouse. Thirty minutes after the dye/antigen injection, the mice were killed; the skin of the abdomen was inverted, and observed for visible bluing. Results were scored as positive if the bluing of the skin at the injection sites was greater than 3 mm in any cross-section diameter.

### Histopathological analysis

Segments of small intestine (jejunum) were embedded in paraffin after fixation in 10% neutral-buffered formaldehyde (formaldehyde, 3.7%; sodium phosphate monobasic, 0.4%; sodium phosphate dibasic anhydrous, 0.65%). Five-micrometer sections were stained with hematoxylin and eosin (H&E). The morphology of the small intestine was photographed using a Nikon Eclipse 80i image-processing system (Nikon Inc., Melville, NY, USA).

### Measurement of RP-specific IgE in sera

Rice protein-specific IgE was assayed by enzyme-linked immunosorbant assay (ELISA). Plates (Nunc-Immunoplate; PolySorp, Roskilde, Denmark) were coated with 100 μg/ml rice protein extract diluted in 50 μl carbonate buffer (pH 9.6) at 4°C for overnight. Plates were then washed 3 times with 200 μl washing buffer and blocked with 75 μl 1% bovine serum albumin (BSA) in PBS for one hour at 37°C. After washing for 3 times, 50 μl undiluted serum samples were added to the plates and incubated overnight at 4°C. Following incubation, plates were washed, and 50 μl of 1:1000 diluted biotin anti-mouse IgE (BioLegend Corp., San Diego, CA, USA) was added to each well. The plates were further incubated for one hour at 37°C. After 3 washes, 50 μl of 1:2000 diluted horseradish peroxidase (HRP)-conjugated streptavidin (BioLegend) was added and incubated for another hour at room temperature. The reactions were developed with 3,3',5,5'-tetramethylbenzidine liquid substrate (Sigma-Aldrich) for 30 minutes at room temperature, stopped with the addition of 1 N H_2_SO4, and optical density were taken at 450 nm wavelength.

### Statistics

Statistical significance (P < 0.05) of differences between individual mice groups was assessed by Student's t-test and one-way ANOVA with the help of Grpahpad Prism 5 (GraphPad Software, Inc. San Diego, CA). Data were expressed as mean ± SD.

## Results

### Rice proteins (RP) extraction and electrophoresis

**Figure 2:**
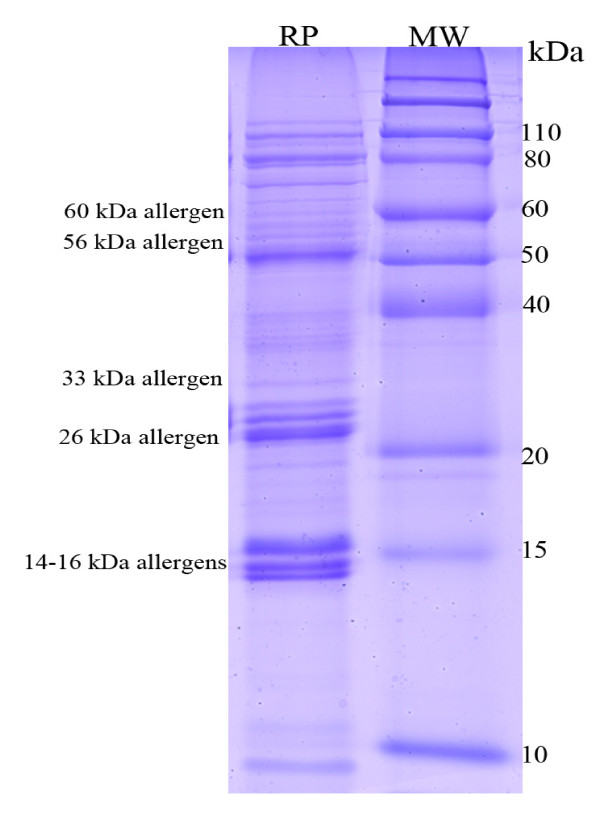
**SDS-PAGE profile of rice seeds proteins including rice major allergens**. Rice proteins were resolved and distinguished upon 12.5% polyacrylamide gel and stained by Coomassie Blue. Lane 1, rice protein (RP); lane 2, Protein molecular weight standards (MW).

Aiming at introducing total raw rice allergens to mouse, our protocol for RP extraction was designed to extract rice proteins to the largest extent. The average recovery efficience of our prototol for four major precipitated rice protein components (albumin, globulin, glutelin, and prolamin) varied from 88.1% to 91.0% in three independent experiments. The SDS-PAGE pattern of RP showed a large variety of proteins, ranging from 10 kDa to 110 kDa (Figure 2), including 14-16, 26, 33, 56, and 60 kDa allergens [17-20]. This raw rice seeds flour was quantitated and the ectracted total RP was detected and applied to BALB/c mice sensitization and challenge protocol, as described above.

### Hypersensitivity symptom induced by i.g. challenge

**Figure 3:**
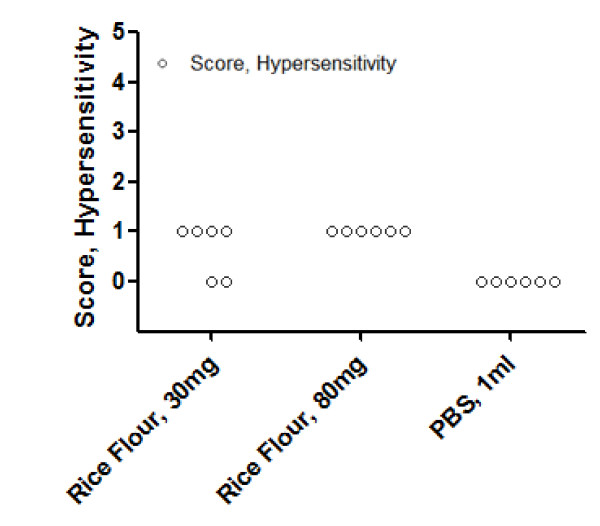
**Symptom scores of hypersensitivity**. BALB/c mice (n = 6) experienced in the fifth intragastric challenge. Symptom scores were recorded 1-1.5 h after challenge as described before. Two observers performed this analysis blinded and in duplicate.

At day 18, after the fifth intragastric challenge, all mice in the higher dose RP challenged group and four out of six mice in the lower dose group showed hypersensitivity reactions. No allergic reactions were detected among the PBS sham-sensitized mice (Figure 3).

### Vascular leakage after i.g. challenge

**Figure 4:**
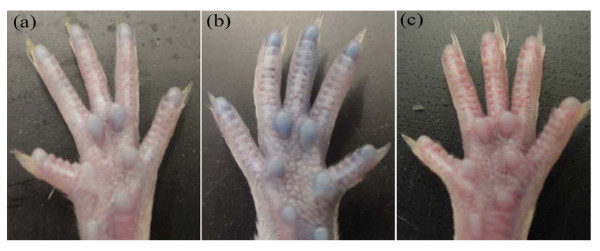
**Rice antigen induced vascular leakage**. Footpads photographed after Evan's Blue Dye/antigen administration. Bluish coloring of the footpads can be detected in (a, b) mouse daily sensitized with 30 or 80 mg rice seeds flour, respectively. (c) PBS sham-sensitized. This part was assayed in duplicates.

The histamine release led to increased vascular permeability, which is regarded as a hallmark of hypersensitivity reaction. Figure 4 presents the bluish coloring of the footpads of challenged mice, which were unnoticeable in sham-sensitized mice after i.g. challenge.

### PCA reactions

Based on the evidence that IgE levels account for most of the rice elicited hypersensitivity and are associated with the severity of anaphylaxis [19], we performed PCA assay to confirm the presence of rice antigen specific IgE in sera (Table 1). IgG1 specific hypersensitivity reactions were eliminated through PCA positive bluing demonstrated by naïve mice injected with unheated RP-immune sera but negative bluing with the heated one.

**Table 1:** PCA reactions after injection of undiluted pooled sera

Donor sensitizaion	Heat inactivation	Diameter (mm) mean ± SD	Positive reaction sites n/total
RP, 30 mg	-	5.32 ± 0.83*	12/12

RP, 80 mg	-	6.96 ± 0.35***	12/12

RP, 80 mg	+	0.13 ± 0.10	0/6

PBS		0.12 ± 0.10	0/12

Pooled sera from mice sensitized with RP were injected intradermally into naïve BALB/c mice (n = 2 to 4) including three antigen and one control injection sites for each mouse. Injection of PBS served as sham control. A positive reaction refers to the skin at the injection sites with bluing greater than 3 mm in diameter. * P < 0.01 versus PBS sham control or heated immune sera. ** P < 0.01 versus sera from 30 mg RP daily sensitized mice group. All detections were repeated twice.

### Characterization of Intestinal Pathology

**Figure 5:**
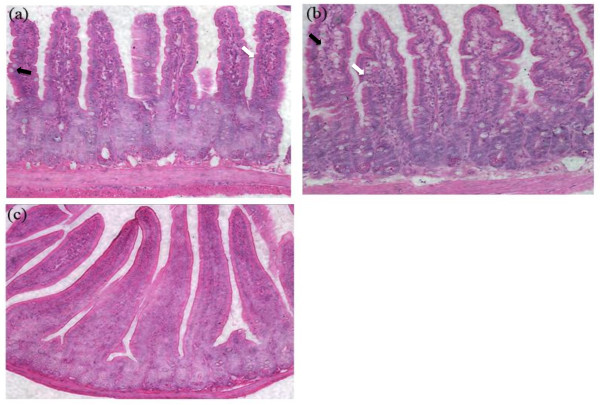
**Ingestion of rice allergens causes histological alterations**. Villi edema, lymphocyte infiltration (white arrows), goblet cells hyperplasia (dark arrow) are shown in the small intestine of BALB/c mice. (a, b) mouse daily sensitized with 30 or 80 mg rice seeds flour, respectively, (c) control mouse with PBS daily sham-sensitization. The H & E stained sections were photographed through a microscope with 20 times magnification.

Histologic examination of the jejunum segments revealed villi edema, lymphocytes infiltration and goblet cells hyperplasia in mice sensitized with 30 mg or 80 mg rice flour and challenged with 30 mg rice total protein (Figure 5A, B vs Figure 5C).

### RP-specific IgE in sera after intragastric sensitization

**Figure 6:**
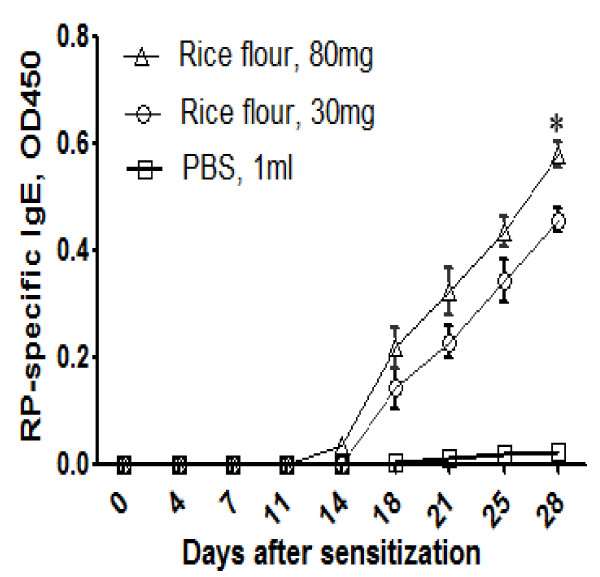
**Dose-response and time dependency of RP-specific IgE by ELISA**. Sera from different groups of mice (n = 5) with the sensitization indicated. The results are presented as mean ± SD. * rice flour, 80 mg versus rice flour, 30 mg, P < 0.01. All samples were assayed in duplicates.

In order to better characterize the RP-specific IgE production during the course of murine rice allergy development, we further detected the serum RP-IgE level before every time point of challenge by ELISA. After the fifth challenge, a significant increase in RP-specific IgE can be observed from day 18 (Figure 6). Mice sensitized with larger dose (80 mg/day) of raw rice seeds flour developed more antigen-specific IgE. RP-specific IgE production in our mice model exhibited both a dose-dependent and time-dependent relationships.

## Discussion

Rice is a staple crop and ingredient consumed worldwide. However, the prevalence of rice allergy makes it an important health issue in many countries, especially for those that treat rice as a major diet component [21]. Under several circumstances, the ingestion or inhalation of rice or rice related products result in allergic reactions in atopic individuals, such as those suffering from rhinitis, urticaria, dermatitis, gastrointestinal symptom or asthma [2-10]. A group of rice allergens including 14-16, 26, 33, 56, and 60 kDa proteins of rice seeds have been identified and proved to be IgE-eliciting *in vivo *[17-20]. Unlike many other food allergies, there is a dearth of information regarding the molecular mechanisms of rice hypersensitivity reactions largely due to the lack of a validated *in vivo *model for studying the physiological and immunological mechanisms of the allergic responses elicited by rice.

Here we present a murine model of rice allergy in BALB/c mice through daily oral sensitization and multiple challenges. Our model demonstrated an IgE-mediated hypersensitivity reaction in mice. In almost all of the experimental mice, the symptoms of hypersensitivity were apparent 1-1.5 h after the fifth i.g. challenge. Symptoms of hypersensitivity included scratching and rubbing around the nose and head, and increased vascular leakage. Elevated RP-specific IgE levels in the sensitized mice and the positive PCA reactions further supported the relevance of the rice allergy mouse model. The phenomenon that the rice allergy caused jejunum inflammation has also been evaluated with the sign of goblet cells hyperplasia in the small intestine, which is consistent with previous reports of food allergy in mice [22,23]. However, the current study is limited in discriminating the contribution between rice flour feeding or extracted rice flour protein challenges leading to the observed sensitization. Instead, we mainly described a simple, robust and feasible mouse model for evaluation of the rice or other cereal food allergy and possibly for further systematic study of the pathomechanisms of the rice allergy. Moreover, the Th1/Th2 cell-mediated immune responses and other immunobiological patterns have not been addressed in our studies.

There was an increase of rice allergy prevalence in the past decades. Among those reported cases, most were isolated clinical observations of allergenic reactions through contact or ingestion of rice. Alternatively, cross-reactivity of rice with other foods had also been demonstrated in several patients [24,25]. The murine model of food allergy, which is regarded as a very useful tool to mimic the allergic reactions in human, has gained promising roles for identification and characterization of proteins responsible in allergy, to better understand the underlying pathogenesis of allergic reactions to food, and for future exploration of new therapeutic approaches [26,27].

Mouse models for different allergens have been published. They could be classified into two major approaches: (i) adjuvant-based or adjuvant-free [28-31], and (ii) sensitized by the intragastric administration or not [32-34]. In the present study, we sensitized and challenged BALB/c mice orally without adjuvant trying to mimic the natural way of human daily rice consumption. Moreover, taking into consideration that rice has an *in vivo *immunogenic capacity through ingestion [10,35], we developed this adjuvant free i.g. sensitization protocol to evaluate the allergenicity of rice. Considering the insoluble property of the prolamin and glutelin fractions of rice in PBS, a relative high dose of total rice protein was used in our protocol to challenge the mice through the i.g. route.

In general, it is believed that high dose of antigen will induce tolerance [12,36]. A peanut allergy mouse model study showed that oral administration of 100 mg peanut protein had a significant effect on reducing the level of peanut-specific IgG, IgE, IgG1 and IgG2a [37]. In this study, we employed 30 mg or 80 mg rice seeds flour for sensitization and 30 mg extracted rice flour proteins for challenge. A reason to do so is that both ground rice seeds flour and extracted rice flour proteins contain a mixture of components. Rice allergens comprise a relatively small part of the total rice seeds proteins and the prolamin and glutelin portion are insoluble in PBS. Another concern is that during the oral administration of antigen, the effective dose for allergenic responses to food antigen increased mainly because the reaction of food proteins to IgE decreased through the gastric digestion [38].

## Conclusions

In conclusion, our data demonstrated the efficacy of applying oral administrative method to elicit hypersensitivity in BALB/c mice, in the absence of adjuvant. We described the time- and dose-dependent responses of rice allergy as indicated by the elevated RP-specific IgE level together with the results from passive cutaneous anaphylaxis assay. Furthermore, our experiments which included multiple identified rice seeds allergens and a large fraction of rice seeds proteins during the course of sensitization, may provide a more holistic view on the allergenicity of rice. This model may be useful in future studies for the underlying mechanisms of allergy caused by rice or other cereals, the evaluation of allergenic candidates in rice or other cereals and the development of immunotherapy in allergy-related diseases.

## Competing interests

The authors declare that they have no competing interests.

## Authors' contributions

XWC and MCF designed the study. XWC carried out the experiments, and prepared the manuscript. KWKL coordinated the study, helped with the experiments and critically revised the manuscript. FY contributed ideas in protein extraction and mouse model establishment. SSMS and MCF conceived, coordinated the study and critically revised the manuscript. All authors read and approved the final manuscript.

## Pre-publication history

The pre-publication history for this paper can be accessed here:

http://www.biomedcentral.com/1471-230X/11/62/prepub
